# 875. Guess Who’s Back: The Return of Seasonal Respiratory Viruses in the State of Wisconsin and Associated Changes in Antibiotic Prescribing for Respiratory Complaints

**DOI:** 10.1093/ofid/ofac492.068

**Published:** 2022-12-15

**Authors:** Alexander Lepak, Lindsay Taylor, Lucas Schulz, Erika Hanson, Jonathan Temte

**Affiliations:** University of Wisconsin School of Medicine and Public Health, Madison, WI; University of Wisconsin School of Medicine and Public Health, Madison, WI; UW Health, Madison, Wisconsin; Wisconsin State Laboratory of Hygiene, Madison, Wisconsin; University of Wisconsin School of Medicine and Public Health, Madison, WI

## Abstract

**Background:**

Return of infections caused by common respiratory viruses (RV) may be expected with the relaxation and/or removal of infection prevention measures instituted during the COVID-19 pandemic. Therefore, we examined respiratory virus detection (RVD) rates in Wisconsin during the COVID-19 period, and whether ambulatory antibiotic prescribing for respiratory complaints has increased with return of typical RVs.

**Methods:**

The Wisconsin State Laboratory of Hygiene’s Viral Surveillance program collects RVD data from >130 laboratories across the state for influenza (FLU), respiratory syncytial virus (RSV), seasonal coronaviruses (sCOR), parainfluenza virus (PARA), enteroviruses/rhinoviruses (E/R), and human metapneumovirus (hMPV). Data were collected from 1/1/2015 to 4/30/2022. Antibiotic prescribing for ambulatory care patients presenting with respiratory complaints was collected from our EHR, which utilizes a required order form for all ambulatory antibiotic prescriptions. Statistical analysis was performed using Mann-Whitney Rank Sum and Spearman’s rank correlation.

**Results:**

In the first year after COVID-19 onset, E/R and sCOR were detected at low levels while other RVs were essentially nil. After 4/2021, when infection prevention measures (i.e. mask mandates) were significantly relaxed or removed, RVDs increased for all viruses. At present, RVDs have returned to typical rates (except of FLU, Fig. 1) and seasonality variation (except of RSV, Fig. 1). Antibiotic prescribing for respiratory complaints has increased 57% in this period (3.5 to 5.5 prescriptions/1000 encounters, Fig. 2) and continues to trend up with RV activity. Prescribing rates are strongly correlated with RVD rates, but most strongly correlated with non-FLU, non-RSV RVD rates (Spearman correlation 0.71).

The dashed vertical line represents relaxation/discontinuation of public infection prevention measures (e.g. masking).

The dashed vertical line represents relaxation/discontinuation of public infection prevention measures (e.g. masking).

* Parainfluenza virus, human metapneumovirus, enterovirus/rhinovirus, or seasonal coronavirus.

**Conclusion:**

In general, RVs have returned to pre-pandemic rates with seasonality. Interestingly, this return was associated with the relaxation/removal of infection prevention measures in the second year post-COVID-19 onset, and does not appear to be impacted by ongoing COVID-19 waves. Antibiotic prescribing in ambulatory care continues to be highly associated with RV activity, indicating this should remain a high priority of ambulatory stewardship education and intervention.

**Disclosures:**

**All Authors**: No reported disclosures.

**Figure 1.**
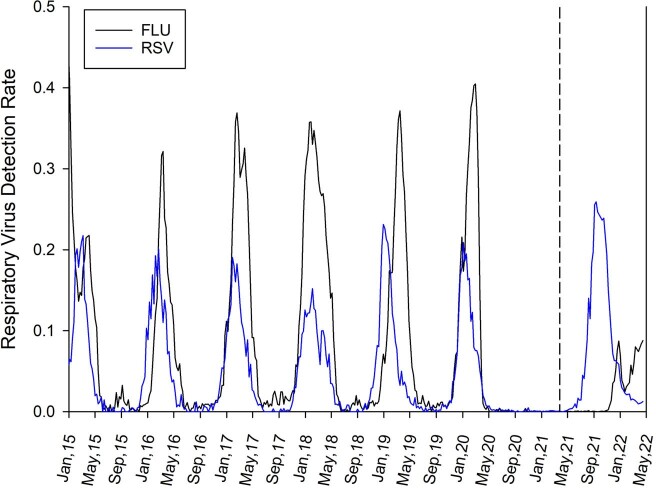
Respiratory virus detection rate over time for influenza (FLU) and respiratory syncytial virus (RSV).

**Figure 2.**
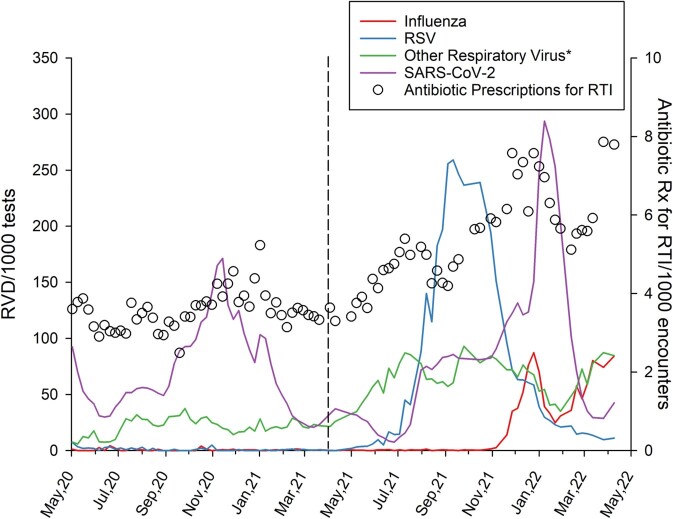
Respiratory virus detection rates and changes in antibiotic prescribing for respiratory tract infections in ambulatory care clinics.

